# Cognitive biases associated with medical decisions: a systematic review

**DOI:** 10.1186/s12911-016-0377-1

**Published:** 2016-11-03

**Authors:** Gustavo Saposnik, Donald Redelmeier, Christian C. Ruff, Philippe N. Tobler

**Affiliations:** 1 Department of Economics, University of Zurich, Zürich, Switzerland; 2 Stroke Program, Department of Medicine, St Michael’s Hospital, University of Toronto, Toronto, M5C 1R6 Canada; 3 Institute for Clinical Evaluative Sciences (ICES), Toronto, Canada; 4 University of Zurich, 9 Blumplistrasse, Zurich, (8006) Switzerland

**Keywords:** Decision making, Cognitive bias, Personality traits, Cognition, Physicians, Case-scenarios, Systematic review

## Abstract

**Background:**

Cognitive biases and personality traits (aversion to risk or ambiguity) may lead to diagnostic inaccuracies and medical errors resulting in mismanagement or inadequate utilization of resources. We conducted a systematic review with four objectives: 1) to identify the most common cognitive biases, 2) to evaluate the influence of cognitive biases on diagnostic accuracy or management errors, 3) to determine their impact on patient outcomes, and 4) to identify literature gaps.

**Methods:**

We searched MEDLINE and the Cochrane Library databases for relevant articles on cognitive biases from 1980 to May 2015. We included studies conducted in physicians that evaluated at least one cognitive factor using case-vignettes or real scenarios and reported an associated outcome written in English. Data quality was assessed by the Newcastle-Ottawa scale. Among 114 publications, 20 studies comprising 6810 physicians met the inclusion criteria. Nineteen cognitive biases were identified.

**Results:**

All studies found at least one cognitive bias or personality trait to affect physicians. Overconfidence, lower tolerance to risk, the anchoring effect, and information and availability biases were associated with diagnostic inaccuracies in 36.5 to 77 % of case-scenarios. Five out of seven (71.4 %) studies showed an association between cognitive biases and therapeutic or management errors. Of two (10 %) studies evaluating the impact of cognitive biases or personality traits on patient outcomes, only one showed that higher tolerance to ambiguity was associated with increased medical complications (9.7 % vs 6.5 %; *p* = .004). Most studies (60 %) targeted cognitive biases in diagnostic tasks, fewer focused on treatment or management (35 %) and on prognosis (10 %). Literature gaps include potentially relevant biases (e.g. aggregate bias, feedback sanction, hindsight bias) not investigated in the included studies. Moreover, only five (25 %) studies used clinical guidelines as the framework to determine diagnostic or treatment errors. Most studies (*n* = 12, 60 %) were classified as low quality.

**Conclusions:**

Overconfidence, the anchoring effect, information and availability bias, and tolerance to risk may be associated with diagnostic inaccuracies or suboptimal management. More comprehensive studies are needed to determine the prevalence of cognitive biases and personality traits and their potential impact on physicians’ decisions, medical errors, and patient outcomes.

**Electronic supplementary material:**

The online version of this article (doi:10.1186/s12911-016-0377-1) contains supplementary material, which is available to authorized users.

## Background

Medical errors occur in 1.7-6.5 % of all hospital admissions causing up to 100,000 unnecessary deaths each year, and perhaps one million in excess injuries in the USA [1, 2]. In 2008, medical errors cost the USA $19.5 billion [3]. The incremental cost associated with the average event was about US$ 4685 and an increased length of stay of about 4.6 days. The ultimate consequences of medical errors include avoidable hospitalizations, medication underuse and overuse, and wasted resources that may lead to patients’ harm [4, 5].

**Fig. 1:**
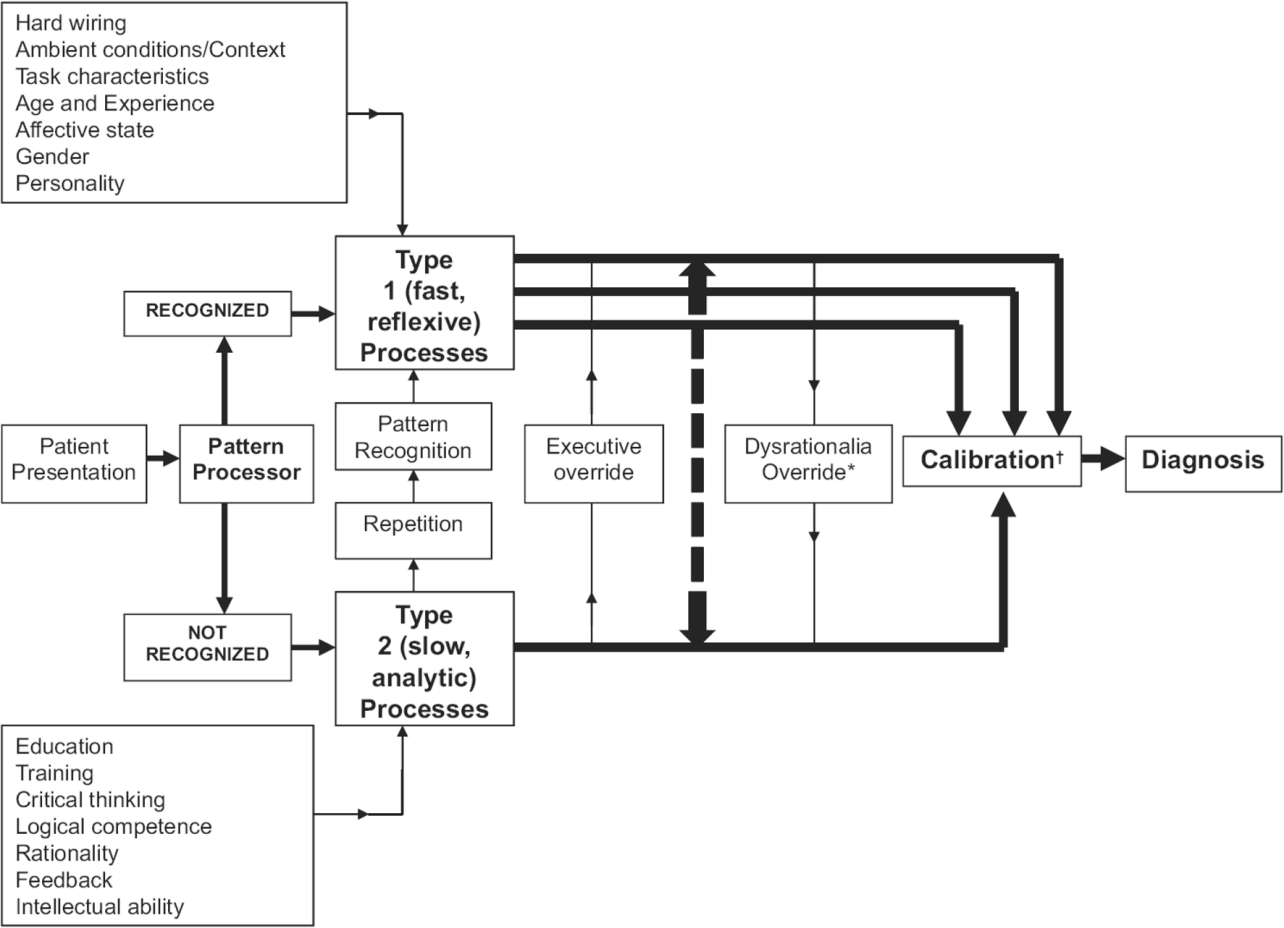
A model for diagnostic reasoning based on dual-process theory (from Ely et al. with permission).[9] System 1 thinking can be influenced by multiple factors, many of them subconscious (emotional polarization toward the patient, recent experience with the diagnosis being considered, specific cognitive or affective biases), and is therefore represented with multiple channels, whereas system 2 processes are, in a given instance, single-channeled and linear. System 2 overrides system 1 (executive override) when physicians take a time-out to reflect on their thinking, possibly with the help of checklists. In contrast, system 1 may irrationally override system 2 when physicians insist on going their own way (e.g., ignoring evidence-based clinical decision rules that can usually outperform them). Notes: Dysrationalia denotes the inability to think rationally despite adequate intelligence. “Calibration” denotes the degree to which the perceived and actual diagnostic accuracy correspond

Kahneman and Tversky introduced a dual-system theoretical framework to explain judgments, decisions under uncertainty, and cognitive biases. System 1 refers to an automatic, intuitive, unconscious, fast, and effortless or routine mechanism to make most common decisions (Fig. 1). Conversely, system 2 makes deliberate decisions, which are non-programmed, conscious, usually slow and effortful [6]. It has been suggested that most cognitive biases are likely due to the overuse of system 1 or when system 1 overrides system 2 [7–9]. In this framework, techniques that enhance system 2 could counteract these biases and thereby improve diagnostic accuracy and decrease management errors.

Concerns about cognitive biases are not unique to medicine. Previous studies showed the influence of cognitive biases on decisions inducing errors in other fields (e.g., aeronautic industry, factory production) [10, 11]. For example, a study investigating failures and accidents identified that over 90 % of air traffic control system errors, 82 % of production errors in an unnamed company, and 50–70 % of all electronic equipment failures were partly or wholly due to human cognitive factors [10]. Psychological assessments and quality assessment tools (e.g. Six Sigma) have been applied in many sectors to reduce errors and improve quality [12–15].

The health sector shares commonalities with industrial sectors including vulnerability to human errors [11, 14]. Therefore, a better understanding of the available evidence on cognitive biases influencing medical decisions is crucial. Such an understanding is particularly needed for physicians, as their errors can be fatal and very costly. Moreover, such an understanding could also be useful to inform learning strategies to improve clinical performance and patient outcomes, whereas literature gaps could be useful to inform future research.

In the last three decades, we learned about the importance of patient- and hospital-level factors associated with medical errors. For example, standardized approaches (e.g. Advanced Trauma Life Support, ABCs for cardiopulmonary resuscitation) at the health system level lead to better outcomes by decreasing medical errors [16, 17]. However, physician-level factors were largely ignored as reflected by reports from scientific organizations [18–20]. It was not until the 1970s that cognitive biases were initially recognized to affect individual physicians’ performance in daily medical decisions [6, 21–24]. Despite these efforts, little is known about the influence of cognitive biases and personality traits on physicians’ decisions that lead to diagnostic inaccuracies, medical errors or impact on patient outcomes. While a recent review on cognitive biases and heuristics suggested that general medical personnel is prone to show cognitive biases, it did not answer the question whether these biases actually relate to the number of medical errors in physicians [25].

In the present (primarily narrative) systematic review, we therefore reviewed the literature reporting the existing evidence on the relation between cognitive biases affecting physicians and medical decisions. Under the concept of cognitive biases, we also included personality traits (e.g. aversion to risk or ambiguity) that may systematically affect physicians’ judgments or decisions, independent of whether or not they result in immediate medical errors. Over 32 types of cognitive biases have been described [26]. Importantly, some of these may reflect personality traits that could result in choice tendencies that are factually wrong, whereas others reflect decisions that are potentially suboptimal, although there is no objectively “correct” decision (e.g. risk aversion, tolerance to ambiguity). Both of these factors were included here.

Our review has four objectives: 1) to identify the most common cognitive biases by subjecting physicians to real world situations or case-vignettes, 2) to evaluate the influence of cognitive biases on diagnostic accuracy and medical errors in management or treatment, 3) to determine which cognitive biases have the greatest impact on patient outcomes, and 4) to identify literature gaps in this specific area to guide future research. After addressing these objectives, we conclude by highlighting the practical implications of our findings and by outlining an action plan to advance the field.

## Methods

### Data sources

We conducted a literature search of MEDLINE and the Cochrane Library databases from 1980 to May 2015 by using a pre-specified search protocol (Additional file 1). We used a permuted combination of MeSH terms as major subjects, including: “medical errors”, “bias”, “cognition”, “decision making”, “physicians”, and “case-vignettes” or “case-scenarios”. In-line with the learning and education literature, case-vignettes, clinical scenarios or ‘real world’ encounters are regarded as the best simple strategy to evaluate cognitive biases among physicians [27]. In addition, this approach has also the advantage of facilitating the assessment of training strategies to ameliorate the influence of cognitive biases on medical errors. We therefore restricted our sample to studies that used case-vignettes or real-world encounters.

**Fig. 2:**
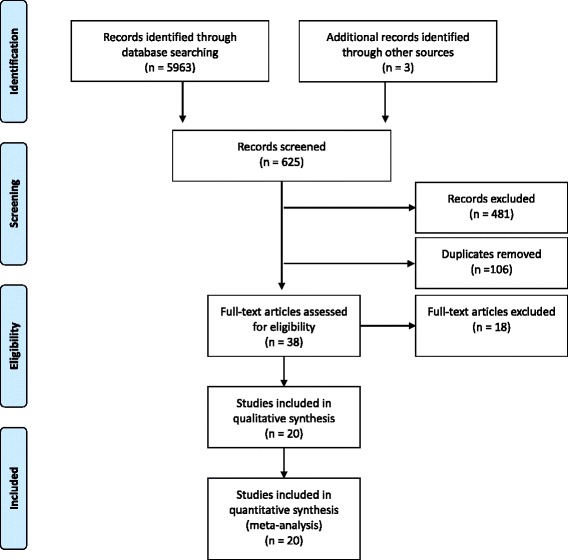
PRISMA flow diagram

Results of the combination of search terms are listed in the Additional file 1. We also completed further searches based on key words, and reviewed references from previously retrieved articles. All articles were then combined into a single list, and duplicates (*n* = 106) were excluded (Fig. 2).

#### Study selection

Candidate articles examining cognitive biases influencing medical decisions were included for review if they met the following five inclusion criteria: First, the study was conducted on physicians. Second, at least one outcome measure was reported. Third, at least one cognitive factor or bias was investigated and defined a priori. Fourth, case-vignettes or real clinical encounters were used [28]. Fifth, the study was written in English. We analyzed the number of articles that fulfilled our inclusion criteria on each cognitive factor or bias, methodological aspects, and the magnitude of effect (as prevalence or odds ratios) on diagnostic or therapeutic decisions. We excluded studies that were not the primary source. We analyzed the original data as reported by the authors. Studies not providing raw data were also excluded (e.g. review articles, letters to Editors).

A recent systematic review was focused on medical personnel in general rather than physicians, and therefore included a different set of studies in their analysis than those that are of interest when considering the impact of cognitive biases on physicians’ medical decision-making and medical errors (the focus of the current study) [25].

### Data extraction

We extracted data according to the Preferred Reporting Items for Systematic Reviews and Meta-Analyses (PRISMA) statement (Fig. 2) [29]. Two reviewers (GS, librarian) assessed titles and abstracts to determine eligibility. Data were extracted using standardized collection forms. Information was collected on country of origin, study design, year of publication, number of studied cognitive biases, population target (general practitioners, specialists, residents), decision type (e.g. diagnosis, treatment, management), unadjusted vs. adjusted analysis (for measured confounders, such as age, years of training, expertise), type of outcome (see below), data quality, and summary main findings. We also included descriptive elements (attributes) of the medical information provided for each case-scenario. The main outcomes were any form of medical error [26, 30], including: underuse or overuse of medical tests, diagnostic accuracy, lack of prescription or prescription of unnecessary medications, outcomes of surgical procedures, and avoidable hospitalizations.

#### Data quality

We used the Newcastle-Ottawa Scale (NOS) to assess the quality of studies (see Additional file 2) [31]. The NOS is a quality assessment tool for observational studies recommended by the Cochrane Collaboration [32]. It assigns one or two points for each of eight items, categorized into three groups: the selection of the study groups; the comparability of the groups; and the ascertainment of the outcome of interest. Previous studies defined NOS scores as: 7–9 points considered as high quality, 5–6 as moderate quality, and 0–4 as low quality [33]. For example, studies that do not provide a description of the cohort, ascertainment of the exposure, adjustment for major confounders, or demonstration that the outcome of interest was not present at the beginning of the study were ranked as low quality [31].

## Results

We identified 5963 studies for the combination of MESH terms “decision making” and “physicians”. Of these, 114 fulfilled the selection criteria and were retrieved for detailed assessment. Among them, 38 articles used case-vignettes or real case scenarios in physicians (Fig. 2). Combinations of other search terms are shown in the Additional file 1: Table S1. Twenty studies comprising 6810 physicians (median 180 per study; range: 36–2206) met the inclusion criteria (Fig. 2) [30, 34–52].

In 55 % (*n* = 11) of the retained studies, results were adjusted for confounders, such as age, gender, level of training (see Additional file 1 for further details). Importantly, only five (25 %) studies used clinical guidelines as the framework to determine diagnostic or treatment errors, illustrating the scarcity of research on evidence-based decision making (e.g. GRADE: decisions based on levels of evidence provided by randomized trials, meta-analysis, etc).

### Population target

Eight (40 %) studies included residents, six (30 %) studies included general practitioners, six (30 %) studies included internists, three (15 %) studies included emergency physicians and seven (35 %) studies included other specialists (Table 2). Ten (50 %) studies were conducted in the USA. Only six (30 %) studies classified errors based on real life measures, such as patient encounters, pathological images or endoscopic procedures, whereas the remaining 14 used narrative case-vignettes. Studies included a wide variety of medical situations, most commonly infections (upper respiratory tract, urinary tract) and cardiovascular disease (coronary disease, cerebrovascular disease) (Table 1). In summary, the included studies covered a wide range of medical conditions and participants.

**Table 1:** Characteristics of studies included in the systematic review

Author	Year of publication	Country	Number participants	Methods	Clinical problem	Type of decision	Cognitive bias (n)	Type of cognitive bias	Data quality*
Redelmeier	1995	Canada	639	Survey	Ostoearthritis, TIA	Management and Treatment	1	Multiple alternative/Decoy bias	5
Ross	1999	UK	407	Survey	Depression	Treatment and management	1	Outcome bias	6
Graber	2000	USA	232	Survey	Headache, abdominal pain, depression	Diagnosis	1	Information bias	4
Sorum	2003	USA, France	65	Survey	Prostate cancer	Diagnosis	1	risk aversion	4
Baldwin	2005	USA	46	Experimental	Brochiolitis	Management	2	risk aversion, Ambiguity tolerance	5
Friedman	2005	USA	216	Survey	NR	Diagnosis	1	Overconfidence	4
Reyna	2006	USA	74	Survey	Unstable angina	Diagnosis and management	1	risk aversion	5
Bytzer	2007	Denmark	127	Video-cases	Reflux, epigastric pain	Diagnosis	1	Infromation bias	4
Dibonaventura	2008	USA	2206	Survey	Immunization	Treatment	2	omissions and naturalness bias	4
Mamede	2010	Netherlands	36	Experiment	Hepatitis, IBD, MI, Wernicke, Pneumonia, UTI, Meningitis	Diagnosis	1	Availability, Reflective reasoning	5
Mamade	2010	Netherlands	84	Survey	Aortic dissection, pancreatitis, hepatitis, pericarditis, hyperthiroidism, sarcoidosis, lung cancer, pneumonia, claudication, bacterial endocarditis	Diagnosis	1	Deliveration without attention	3
Gupta	2011	USA	587	Survey	Abdominal pain, headache, trauma, asthma, chest pain	Diagnosis	1	Outcome bias	6
Perneger	2011	Switzerland	1439	Survey	HIV infection	Treatment-Prognosis	1	Framing effect	4
Stiegler	2012	USA	64	Delphi and 38 simulated encounters	anaphylaxis, malignant hyperthermia, difficult airway, and pulmonary embolism	Treatment and management	10	anchoring, availability bias, premature closure, feedback bias, framing effect, confirmation bias, omission	4
Ogdie	2012	USA	41	Narratives	NR	Diagnosis	9	Anchoring, availability, framing effect, blind obedience, confirmation	3
Meyer	2013	USA	118	Survey	Abdominal pain, headache and rash, fever and arthralgias	Diagnosis	1	Overconfidence	4
Crowley	2013	International	71	Pathology cases	Vesicular and diffuse dermatitides	Diagnosis	8	anchoring, availability bias, confirmation bias, overconfidence	4
Saposnik	2013	Canada	111	Case-scenarios from real practice	Stroke	Prognosis	2	Overconfidence, anchoring	5
Msaouel	2014	Greece	153	Survey	Tuberculosis, CAD	Diagnosis	2	Gambler’s and Conjunction fallacy	5
Yee	2014	USA	94	Experimental	Deliveries	Management and Treatment	1	Ambiguity tolerance/aversion	7

*Data quality assessed using the Newcastle-Ottawa acale (NOS)

### Data quality

All studies were designed as cohort studies evaluating cognitive biases. According to the NOS, the majority of studies (*n* = 12, 60 %) were low quality, seven (35 %) studies ranked moderate and only one ranked as high quality [43] (see Additional file 2: Table S2 for details). All studies were classified as representative of the entire population (defined as how likely the exposed cohort was included in the population of physicians).

### Presence of most common cognitive biases (Objective 1)

Our first objective was to evaluate the most common cognitive biases affecting physicians’ decisions. Altogether, studies evaluated 19 different cognitive biases (Table 1 and Additional file 1).

It is important to bear in mind that these studies do not systematically assess each cognitive bias or personality traits. As a result, it is not possible to provide a true estimate of the prevalence of all cognitive biases among physicians. Overall, at least one cognitive factor or bias was present in all studies. Studies evaluating more than two cognitive biases, found that 50 to 100 % of physicians were affected by at least one [39, 50, 52]. Only three manuscripts evaluated more than 5 cognitive biases in the same study, in-line with the narrow scope of most studies [39, 50, 52]. One third of studies (*n* = 6) were descriptive, i.e., they provided the frequency of the cognitive bias without outcome data [36, 37, 39, 44, 48, 51].

**Fig. 3:**
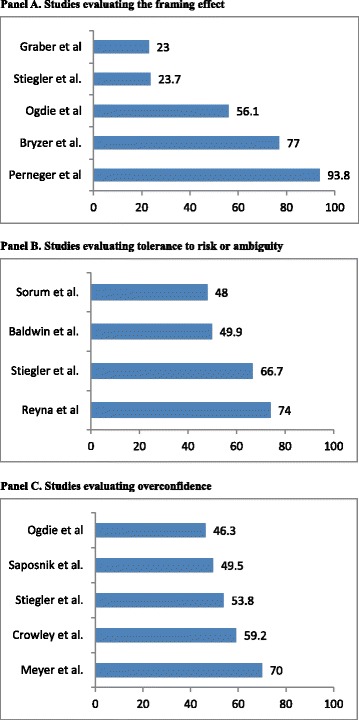
Prevalence of most common cognitive biases as reported by different studies. Numbers represent percentages reflecting the frequency of the cognitive factor/bias. Panel **a** represent the prevalence of the framing effect. Panel **b** represent the prevalence of prevalence of tolerance to risk and ambiguity. Panel **c** represents the prevalence of overconfidence. Overall, overconfidence and low tolerance to risk or ambiguity were found in 50-70 % of participants, whereas a wide variation was found for the framing effect

**Fig. 4:**
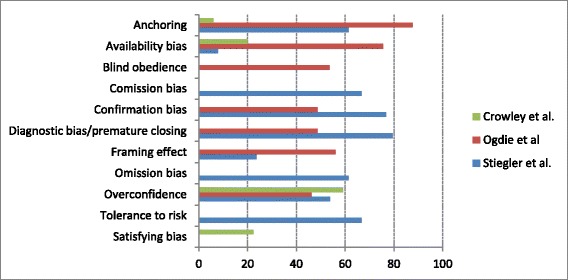
Prevalence of cognitive biases in the top three most comprehensive studies [39, 50, 52] Numbers represent percentages reflecting the frequency of the cognitive bias. Note the wide variation in the prevalence of cognitive biases across studies

The most commonly studied personality trait was tolerance to risk or ambiguity (*n* = 5), whereas the framing effects (*n* = 5) and overconfidence (*n* = 5) were the most common cognitive biases. There was a wide variability in the reported prevalence of cognitive biases (Fig. 3). For example, when analyzing the three most comprehensive studies that accounted for several cognitive biases (Fig. 4), the availability bias ranged from 7.8 to 75.6 % and anchoring from 5.9 to 87.8 %, suggestive of substantial heterogeneity among studies. In summary, cognitive biases may be common and present in all included studies. The framing effect, overconfidence, and tolerance to risk/ambiguity were the most commonly studied cognitive biases. However, methodological limitations make it difficult to provide an accurate estimation of the true prevalence.

### Effect of cognitive biases on medical tasks (Objective 2)

**Fig. 5:**
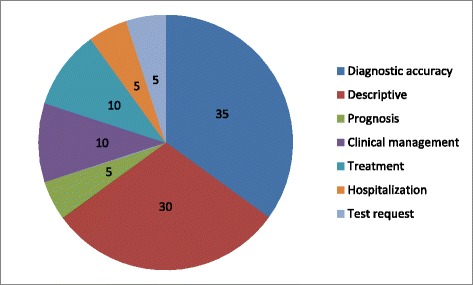
Outcome measures of studies evaluating cognitive biases. Numbers represent percentages. Total number of studies = 20. Note that 30 % of studies are descriptive and 35 % target diagnostic accuracy. Only few studies evaluated medical management, treatment, hospitalization or prognosis

Our second objective concerned the assessment of the influence of cognitive biases on diagnostic, medical management or therapeutic tasks. Most studies (12/20; 60 %) targeted cognitive biases in diagnostic tasks, 7 (35 %) studies targeted treatment or management tasks, and 2 studies (10 %) focused on errors in prognosis. The main measure was diagnostic accuracy in 35 % (7/20) of studies (Fig. 5). Overall, the presence of cognitive biases was associated with diagnostic inaccuracies in 36.5 to 77 % of case-scenarios [30, 35, 40, 42, 45, 52, 53]. A study including 71 residents, fellows, and attending pathologists evaluated 2230 skin biopsies with a diagnosis confirmed by a panel of expert pathologists. Information biases, anchoring effects, and the representativeness bias were associated with diagnostic errors in 51 % of 40 case-scenarios (compared to 16.4 % case-scenarios leading to incorrect diagnoses not related to cognitive biases; *p* = 0.029) [52].

Only seven (35 %) studies provided information to evaluate the association between physicians’ cognitive biases and therapeutic or management errors [38, 41–43, 46, 47, 50]. Five out of the seven (71.4 %) studies showed an association between cognitive biases and these errors [38, 43, 46, 47, 50]. One study showed that overutilization of screening for prostate cancer among healthy individuals was associated with lower aversion to uncertainty (*p* < 0.01) [46]. In another study including 94 obstetricians who cared for 3488 deliveries, better coping strategies (*p* < .015) and tolerance to ambiguity (*p* < .006) were associated with optimal management (reflected by lower instrumental vaginal deliveries) and lower errors [43]. In a study including 32 anesthesiology residents, several cognitive biases (anchoring, overconfidence, premature closure, confirmation bias, etc.) were associated to errors in half of the 38 simulated encounters [50]. Two studies evaluating triage strategies for patients with bronchiolitis and coronary artery disease showed no association between personality traits (e.g. risk aversion or tolerance to uncertainty) and hospital admissions [41, 42].

In summary, our findings suggest that cognitive biases (from one to two thirds of case-scenarios) may be associated with diagnostic inaccuracies. Evidence from five out of seven studies suggests a potential influence of cognitive biases on management or therapeutic errors [38, 43, 46, 47, 50]. Physicians who exhibited information bias, anchoring effects and representativeness bias, were more likely to make diagnostic errors [38, 43, 46, 50].

Further studies are needed to identify what the most common cognitive biases and the most effective strategies to overcome their potential influence of medical tasks and errors.

### Effect of physician’s cognitive biases on patient outcomes (Objective 3)

The third objective of the present study was to determine the impact of cognitive biases on patient outcomes (e.g. avoidable hospitalizations, complications related to a procedure or medication, exposure to unnecessary invasive tests, etc). Only two (10 %) studies provided information to answer this question, both evaluating physicians’ tolerance to uncertainty [41, 43]. In a study evaluating obstetrical practices, higher tolerance to ambiguity was associated with an increased risk of postpartum hemorrhage (9.7 % vs 6.5 %; *p* = .004). The negative effects persisted in the multivariable analysis (for postpartum hemorrhage: OR 1.51, 95 % CI 1.10–2.20 and for chorioamninitis: OR 1.37, 95 % CI 1.10–1.70) [43]. This phenomenon could be explained by overconfidence and underestimation of risk factors associated with maternal infections or puerperal bleeding. On the other hand, a study including 560 infants with bronchiolitis presented to the emergency department cared for by 46 pediatricians showed similar admission rates among physicians with low and high risk aversion or discomfort with diagnostic uncertainty (measured using a standardized tool) [41].

In summary, there too little evidence to make definitive conclusions on the influence of physicians’ personality traits or cognitive biases on patient outcomes.

### Literature gaps and recommendations (Objective 4)

We systematically reviewed gaps in the literature. First, most of the studies (60 %) provided a qualitative definition of cognitive biases based on the interpretation of comments made by participants (e.g. illustrative quotes), lacking a unified and objective assessment tool [39, 50]. Second, the unit of study varies from study to study. For example, some authors report results based on the number of physicians involved in the study, whereas others report the results based on the number of case-scenarios. Third, limited information is currently available on the impact of cognitive biases on evidence-based care, as only 15 % of the studies were based on or supported by clinical guidelines (Table 2). Fourth, only one study evaluated the effect of an intervention (e.g. reflective reasoning) to ameliorate cognitive biases in physicians [35]. Fifth, most studies were classified as low quality according to NOS criteria. However, this scale is regarded as having a modest inter-rater reliability. We need consensus among researchers on the best tools to assess the quality of manuscripts. Sixth, only two studies evaluated the influence of physicians’ biases on patient outcomes. Finally, considering the great majority of studies (85 %) targeted only one or two biases (Table 1), the true prevalence of cognitive biases influencing medical decisions remains unknown.

**Table 2:** Participants, attributes and outcomes of included studies

Author	Type of participants	Number of vignettes or medical cases	Number of attributes	Based on Guidelines	Outcome measure	Type of outcome^a^	Type of analysis	Data quality^b^	Main findings
Redelmeier	GPs and Neurologist	4	10-11	yes	Treatment recommendations	4	unadjusted	5	Multiple options decreased the likelihood of medication prescription for pain and carotid endarterectomy by 26 % and 35 %, respectively
Ross	GPs	3	NA	No	Descriptive	5	adjusted	6	GPs were less likely to arrange a further consultation for female patients than for male patients (OR = 0.55). GPs with a pessimistic belief about depression were less likely to discuss non-physical symptoms or social factors; More experienced GPs were less likely to conduct a physical examination (OR = 0.60).
Graber	GPs	2	8-9	No	Descriptive	1	adjusted	4	GPs were less likely to believe a serious medical condition among patients with history of depression or somatic symptoms
Sorum	GPs	32	5	yes	Probability of ordering a test	4	adjusted	4	PSA were more likely ordered among GPs with discomfort for uncertainty and those who expressed regret.
Baldwin	Pediatric ED physicians	397	NA	No	Admission rates	4	adjusted	5	Risk aversion scores higher for physicians with >15 years of experience. Admissions rates did not differ between high and low risk adverse physicians (31.1 vs 30.1; p = 0.91). Adjusted admission rates did not different between high and low discomfort with uncertainty (32.3 vs 29.7; p = 0.84)
Friedmann	Medical students (72), residents (72), physicians (72)	36 (9)	>20	No	Diagnostic accuracy	5	adjusted	4	Overconfident found in 41 % of residents and in 36 % faculty.
Reyna	GPs and specialists	9	NA	Yes	Diagnostic accuracy and management	6	adjusted	5	Physicians deviated from Guidelines in terms of discharge. GP were more risk averse and less likely to discharge patients. Experts achieved better case-risk discrimination by processing less information
Bytzer	Specialists	5	NA	No	Diagnostic accuracy	6	unadjusted	4	Only 23 % endoscopists gave the same diagnosis for the two identical video-cases. The great majority were affected by prior information bias.
Dibonaventura	Physicians	2	11--12	No	Descriptive	4	unadjusted	4	Naturalness bias present in 40 %, omission bias in 60 % of participants
Mamede	Residents	8	NA	No, confirmed diagnosis	Diagnostic accuracy	5	unadjusted	5	Availability bias increased with years of training. Clinical reasoning ameliorate this bias
Mamade	internal medicine residents (34) and medical students (50)	12	>20	No	Diagnostic accuracy	6	unadjusted	3	Conscious deliberation improved the likelihood of correct diagnosis in physicians, but not in medical students problems were complex, whereas reasoning mode did not matter in simple problems. In contrast, deliberation without attention improved novices’ decisions.
Gupta	ED Physicians	6	>20	No	Descriptive	1	adjusted	6	Outcome bias tends to inflate ratings in the presence of a positive outcome more than it penalizes scenarios with negative ones.
Perneger	GPs and specialists, and patients (1121)	1	5	No	Rating of new drug	6	adjusted	4	Physicians and patients provided higher value to the hypothetical new medication when presented in relative terms. Compared to descriptive information, relative mortality reduction (OR 4.40; 3.05 – 6.34), Number needed to treat (OR 1.79; 1.21 – 2.66), and relative survival extension (OR 4.55; 2.74 – 7.55) had a more positive perception.
Stiegler	Residents (32), Faculty (32)	20	NA	Catalogue of common cases	Management	1	unadjusted	4	1. Developed a cognitive factor/bias catalogue, 2. Top 10 cognitive biases and personality traits: anchoring, availability bias, omission bias, commission bias, premature closure, confirmation bias, framing effect, overconfidence, feedback bias, and sunk cost. 3. Errors perceived by faculty to be important to anesthesiology were indeed observed frequently among trainees in a simulated environment.
Ogdie	Residents	41	NA	No	Descriptive	6	unadjusted	3	Most common biases: anchoring (88 %), availability (76 %), framing effect (56 %), overconfidence (46 %)
Meyer	Physicians	4	6-11	No	Diagnostic accuracy	2	unadjusted	4	Higher confidence was related to decreased requests for additional diagnostic tests (P = .01); higher case difficulty was related to more requests for additional reference materials (P = .01).
Crowley	pathology residents, fellows and staff pathologists	40	NA	No	Diagnostic accuracy	6	unadjusted	4	Overall, biases occurred in 52 % of incorrect cases compared to 21 % correct. Most common biases-Availability (20 %) and satisfying biases (22.5 %) the two most common. All the rest, less than 10 %.
Saposnik	Residents, internists, emergency physicians and Neurologist	10	5-7	No	Probability of death or disability	6	adjusted	5	Higher confidence was not associated with better outcome predictions. 70 % of underestimated the risk of the death or disability, 38 % overestimated death at 30 days.
Msaouel	Residents	2	4, 5	No	Descriptive	1	adjusted	5	Gambler’s fallacy in 46 %, conjunction bias 69 %
Yee	Specialists (Obstetricians)	3488	NA	No	Management	6	adjusted	7	Physicians with a higher tolerance of ambiguity were less likely to deliver patients by operative vaginal delivery (11.8 % vs 16.4 %; p = 0.006). The effect disappeared in the adjusted analysis (OR 0.77, 95 % CI 0.53-1.1)

*NA* not available, *GP* general practitioners

^a^Type of outcome measured: 1 = probability, 2 = rating, 3 = ranking, 4 = yes/no choice, 5 = discrete choice, 6 = more than 2 alternatives

^b^Data quality assessed by the Newcastle-Ottawa Score. See details in the text and Additional file 2

As mentioned, medical errors are common in medical practice [5]. Physicians’ biases and personality traits may explain, at least in part, some medical errors. Given the wide practice variability across medical disciplines, decisions on screening tests, surgical procedures, preventative medications, or other interventions (e.g. thrombolysis for acute stroke, antibiotics for an underlying infection, etc.) may not require the same cognitive abilities it is therefore likely that studies from one discipline cannot be transferred automatically to a different discipline. By extension, physicians’ personality traits (e.g. aversion to ambiguity, tolerance to uncertainty) or cognitive biases (e.g. overconfidence) may not equally influence patient outcomes or medical errors in all disciplines. Time-urgency of the medical decision may be a relevant characteristic. Thus, a discipline-based research approach may be needed. There is scarce information in some disciplines and areas, including anesthesiology (decisions on procedures and anesthetic agents), emergency care, obstetrics and gynecology (e.g. decisions on procedures and primary care on women’s health), endoscopic procedures (e.g. gastrointestinal, uropelvic), neurology (e.g. decision in multiple sclerosis and stroke care).

## Discussion

Early recognition of physicians’ cognitive and biases are crucial to optimize medical decisions, prevent medical errors, provide more realistic patient expectations, and contribute to decreasing the rising health care costs altogether [3, 8, 54]. In the present systematic review, we had four objectives. First, we identified the most commonly reported cognitive biases (i.e., anchoring and framing effects, information biases) and personality traits (e.g. tolerance to uncertainty, aversion to ambiguity) that may potentially affect physicians’ decisions. All included studies found at least one cognitive factor/bias, indicating that a large number of physicians may be possibly affected [39, 50, 52]. Second, we identified the effect of physician’s cognitive biases or personality traits on medical tasks and on medical errors. Studies evaluating physicians’ overconfidence, the anchoring effect, and information or availability bias may suggest an association with diagnostic inaccuracies [30, 35, 40, 42, 45, 52, 53]. Moreover, anchoring, information bias, overconfidence, premature closure, representativeness and confirmation bias may be associated with therapeutic or management errors [38, 43, 46, 47, 50]. Misinterpretation of recommendations and lower comfort with uncertainty were associated with overutilization of diagnostic tests [46]. Physicians with better coping strategies and tolerance to ambiguity could be related to optimal management [43].

For our third objective – identifying the relation between physicians’ cognitive biases and patient’s outcomes- we only had very sparse data: Only 10 % of studies provided data on this area [41, 43]. Only one study showed higher complications (OR 1.51, 95 % CI 1.10–2.20) among patients cared for by physicians with higher tolerance to ambiguity [43]. The fourth and final objective was to identify gaps in the literature. We found that only few (<50 %) of an established set of cognitive biases [26] were assessed, including: overconfidence, and framing effects. Other listed and relevant biases were not studied (e.g. aggregation bias, feedback sanction, hindsight bias). For example, aggregation bias (the assumption that aggregated data from clinical guidelines do not apply to their patients) or hindsight bias (the tendency to view events as more predictable than they really are) both compromise a realistic clinical appraisal, which may also lead to medical errors [18, 26]. More importantly, only 35 % of studies provided information on the association between cognitive biases or personality traits and medical errors [38, 41–43, 46, 47, 50], with scarce information on their impact on patient outcomes, preventing us from making definite conclusions [41, 43]. Furthermore, the quality of the included studies was classified as low to modest according to NOS criteria, as most studies provided limited descriptions of the exposure and research cohort, and none contributed with follow-up data (e.g. sustainability and reliability of the effects or long-term outcomes) (Additional file 2).

When comparing the previous systematic review on patients and medical personnel [25] with ours, some commonalities are apparent. Both reviews agree on the relevance of the topic, identify that a systematic analysis of the impact of cognitive biases on medical decisions is lacking despite substantial work completed in the last two decades [25]. Having a different objective, the authors nicely summarized the number of studies that investigated each cognitive bias either in patients or medical personnel [25]. Similarly, cognitive biases seem to be common among physicians as identified in 80 % (*n* = 51) of studies included in Blumenthal-Barby and Krieger’s review and all selected studies (*n* = 20) evaluating at least one outcome in the present review [25].

However, both studies were not able to provide an accurate estimate of the true prevalence of cognitive biases or personality traits affecting medical decisions in physicians.

On the other hand, our study adds relevant information regarding the influence of cognitive biases particularly in physicians on diagnostic inaccuracies, suboptimal management and therapeutic errors, and patient outcomes. Our first objective allowed the identification of additional biases (e.g. framing effect, decoy effect, default bias) or physician’s personality traits (e.g. low tolerance to uncertainty, aversion to ambiguity), by including 14 further studies. We also completed a systematic quality assessment of each study using a standardized tool and identified gaps related to the influence of cognitive biases on medical errors [31].

### What can be done?

The identification and recognition of literature gaps constitute the first step to finding potential solutions. Increasing awareness among physicians and medical students is an important milestone. A comprehensive narrative review comprising 41 studies on cognitive interventions to reduce misdiagnosis found three main effective strategies: increasing knowledge and expertise, improving clinical reasoning, and getting help from colleagues, experts and tools [55]. First, reflective reasoning counteracts the impact of cognitive biases by improving diagnostic accuracy in second- (OR 2.03; 95 % CI, 1.49–2.57) and first-year residents [OR (odds ratio) 2.31; 95 % CI, 1.89–2.73] [35]. Second, the implementation of tools (e.g. cognitive checklist, calibration) may overcome overconfidence, the anchoring and framing effects (Fig. 5) [8, 9, 56]. Third, heuristics approaches (shortcuts to ignore less relevant information to overcome the complexity of some clinical situations) can improve decision making. As shown by Marewski and Gigerenzer, the identification of three rules (search for predictors to determine their individual importance, stop searching when relevant information was already obtained, and a criteria that specifies how a decision is made) may facilitate prompt decisions and may help physicians to avoid errors in some clinical situations [21, 57, 58].

The inclusion of training in cognitive biases in graduate and postgraduate programs might foster medical education and thereby improve health care delivery [59]. A commitment from academic institutions, scientific organizations, universities, the public, and policy-makers would be needed to reduce a defensive medical practice [60, 61]. An initial step towards this goal may be the ‘Choosing wisely’ strategy [62, 63].

### What are the practical implications of our findings?

As shown, cognitive biases and personality traits may affect our clinical reasoning processes which may lead to errors in the diagnosis, management, or treatment of medical conditions [6, 26]. Errors perceived by faculty to be relevant were indeed observed in 50–80 % of trainees in real practice [50]. Misdiagnosis, mismanagement, and mistreatment are frequently associated with poorer outcomes, which are the most common reasons for patients’ dissatisfaction and medical complaints [54, 64, 65].

Our study has several limitations that deserve comment. First, although we aimed to be as systematic as possible in reviewing the literature, we cannot rule out involuntary omissions. It is also possible that our results may be somewhat limited by the strictness of our inclusion criteria. Second, we were not able to complete a formal meta-analysis due to the diversity of definitions and data reported, and small number of studies evaluating specific cognitive biases. In particular, a limited number of studies evaluated the same constructs. Moreover, across studies we often found a lack (in 30 % of studies) or heterogeneity in the outcome measures, mixed denominators (some studies report their findings based on the number of participants, while others based on case-scenarios) [41, 43, 52], and different scope (e.g. some studies are descriptive, [36, 37, 39, 44, 48, 51] whereas others [7, 30, 35, 42, 43, 47, 50, 52] target diagnostic or therapeutic errors). Third, most studies use hypothetical case-vignettes which may not truly reflect medical decisions in real life. Fourth, the assessment of the number of medical elements included in each case scenario may not be consistent (some were reported by authors and others estimated based on the description of case-scenarios) [35, 40, 51]. Fifth, the use of the NOS to assess the quality of studies has been criticized for having modest inter-rater reliability [66, 67].

Despite the aforementioned limitations, our study reflects the relevance and potential burden of the problem, how little we know about the implications of cognitive biases and personality traits on physicians’ decisions, and their impact on patients-oriented outcomes. Our findings may also increase physicians’ awareness of own personality traits or cognitive biases when counseling or advising patients and their family members that may lead to medical errors. From a health policy perspective, this information would provide additional insights on medically relevant cognitive biases and personality traits that contribute the rising health care costs [3, 68].

## Conclusions

In the present systematic review, we highlighted the relevance of recognizing physicians’ personality traits and cognitive biases. Although cognitive biases may affect a wide range of physicians (and influence diagnostic accuracy, management, and therapeutic decisions), their true prevalence remains unknown.

Thus, substantial gaps limit our understanding of the impact of cognitive biases on medical decisions. As a result, new research approaches are needed. We propose the design of more comprehensive studies to evaluate the effect of physicians’ personality traits and biases on medical errors and patient outcomes in real medical encounters and interventions or using guideline-based case-vignettes. This can be accomplished by identifying physician characteristics, combining validated surveys and experiments commonly used in behavioral economics to elicit several critical personality traits (e.g. tolerance to uncertainty, aversion to risk and ambiguity), and cognitive biases (e.g. overconfidence, illusion of control). Prospective studies evaluating and comparing different training strategies for physicians are needed to better understand and ameliorate the potential impact of cognitive biases on medical decisions or errors. In addition, effective educational strategies are also needed to overcome the effect of cognitive biases on medical decisions and interventions. Together, this information would provide new insights that may affect patient outcomes (e.g. avoidable hospitalizations, complications related to a procedure or medication, request of unnecessary tests, etc) and help attenuate medical errors [3, 68, 69].

## Additional files

Additional file 1: Literature search and definitions of cognitive biases and personality traits. (PDF 101 kb)
Additional file 2: Newcastle-Ottawa assessment tool and data quality. (PDF 76 kb)

## Abbreviations

CI: Confidence intervals
NOS: Newcastle-Ottawa Scale
OR: Odds ratio
PRISMA: Preferred reporting items for systematic reviews and meta-analyses
