# Supplementary material for: Cognitive biases associated with medical decisions: a systematic review
Source: BMC Med Inform Decis Mak. 2016 Nov 3;16:138. doi: 10.1186/s12911-016-0377-1 (PMC5093937; doi:10.1186/s12911-016-0377-1)
Supplement: Additional file 1: — Literature search and definitions of cognitive biases and personality traits. (PDF 101 kb) [file 12911_2016_377_MOESM1_ESM.pdf]

Additional file 1

Statistical Analysis: Chi square tests were used to compare categorical variables when analyzing the association between number of elements in each case scenario and medical errors.

Kruskal-Wallis was used to compare continuous variables when analyzing the number of medical elements and patients' outcomes. The statistical analysis was performed using STATA version 13 (College Station, TX: StataCorp LP). All tests were 2-tailed, and p values <0.05 were considered significant.

Additional descriptive results: The median (interquartile range) number of cognitive distortions evaluated per study was 1 (1-2). The median (interquartile range) number of case-scenarios per study was 9 (4-34). There was no association between the number of cases and the probability of finding medical errors (p=0.17).

The number of medical elements included in each case ranged from 4 to over 20. This information was not available for 9 (45%) of the studies. There was no association between the number of medical elements and the probability of finding medical errors (p=0.62) or affecting patients' outcomes (p=1.0).

Details of search strategy:

1- "Decision making"[MeSH Terms] AND "bias (epidemiology)"[MeSH Terms]

2- "Decision making"[MeSH Terms] AND “Physicians"[MeSH Terms]

3- "Decision making"[MeSH Terms] AND "medical errors"[MeSH Terms]

4- "Decision making"[MeSH Terms] AND "cognition"[MeSH Terms]

5- "Bias (epidemiology) "[MeSH Terms] AND "physicians"[MeSH Terms]

6- "Bias (epidemiology) "[MeSH Terms] AND "medical errors"[MeSH Terms]

7- "Bias (epidemiology) "[MeSH Terms] AND "cognition"[MeSH Terms]

8- "Cognition"[MeSH Terms] AND "physicians"[MeSH Terms]

9- "Cognition"[MeSH Terms] AND "medical errors"[MeSH Terms]

10- "Medical errors"[MeSH Terms] AND "physicians"[MeSH Terms]

11- 9) AND "physicians"[MeSH Terms]

12- 3) AND "physicians"[MeSH Terms]

13- 6) AND "physicians"[MeSH Terms]

14- 4) AND "physicians"[MeSH Terms]

15- 7) AND "physicians"[MeSH Terms]

16- 1) AND "physicians"[MeSH Terms]

17- 9) AND "physicians"[MeSH Terms]

18- 3) AND "case vignettes"[Title/Abstract] OR "case scenario"[Title/Abstract]

19- 6) AND "case vignettes"[Title/Abstract] OR "case scenario"[Title/Abstract]

20- 4) AND "case vignettes"[Title/Abstract] OR "case scenario"[Title/Abstract]

21- 7) AND "case vignettes"[Title/Abstract] OR "case scenario"[Title/Abstract]

22- 1) AND "case vignettes"[Title/Abstract] OR "case scenario"[Title/Abstract]

23- 3) AND "physicians"[MeSH Terms] AND "case vignettes"[Title/Abstract] OR "case scenario"[Title/Abstract]

24- 6) AND "physicians"[MeSH Terms] AND "case vignettes"[Title/Abstract] OR "case scenario"[Title/Abstract]

25- 4) AND "physicians"[MeSH Terms] AND "case vignettes"[Title/Abstract] OR "case scenario"[Title/Abstract]

26- 7) AND "physicians"[MeSH Terms] AND "case vignettes"[Title/Abstract] OR "case scenario"[Title/Abstract]

27- 1) AND "physicians"[MeSH Terms] AND "case vignettes"[Title/Abstract] OR "case scenario"[Title/Abstract]

Definitions of identified cognitive biases and personality traits

Ambiguity aversion: The tendency to avoid options for which missing information makes the probability seems "unknown".
Anchoring: the tendency to perceptually lock onto salient features in the patient’s initial presentation too early in the diagnostic process, and failing to adjust this initial impression in the light of later information.
Ascertainment bias: occurs when a physician’s thinking is shaped by prior expectation (e.g. stereotyping and gender bias).
Availability: the disposition to judge events as being more likely, or frequently occurring, if they readily come to mind. Thus, recent experience with a disease may inflate the likelihood of its being diagnosed. Conversely, if a disease has not been seen for a long time (is less available), it may be underdiagnosed.
Commission bias: results from the obligation toward beneficence, in that harm to the patient can only be prevented by active intervention. It is the tendency toward action rather than inaction. It is more likely in over-confident physicians. Commission bias is less common than omission bias.
Confirmation bias: the tendency to look for confirming evidence to support a diagnosis rather than look for disconfirming evidence to refute it, despite the latter often being more persuasive and definitive.
Conjunction fallacy: The tendency to assume that specific conditions are more probable than general ones.
Framing effect: the tendency to draw different conclusions from the same information, depending on how that information is presented.
Gambler’s fallacy: The tendency to think that future probabilities are altered by past events, when in reality they are unchanged. This results from an erroneous conceptualization of the law of large numbers. For example, "I've flipped heads with this coin five times consecutively, so the chance of tails coming out on the sixth flip is much greater than heads". Another example would be a physician who sees a series of patients with chest pain in clinic or the emergency department, diagnoses all of them with an acute coronary syndrome, and assumes the sequence will not continue.
Multiple alternatives bias (decoy effect): a multiplicity of options on a differential diagnosis may lead to significant conflict and uncertainty. This is a phenomenon whereby consumers will tend to have a specific change in preference between two options when also presented with a third option that is asymmetrically dominated.
Omission bias: the tendency toward inaction and rooted in the principle of non-maleficence. In hindsight, events that have occurred through the natural progression of a disease are more acceptable than those that may be attributed directly to the action of the physician.
Outcome bias: the tendency to opt for diagnostic decisions that will lead to good outcomes, rather than those associated with bad outcomes.
Overconfidence bias: a universal tendency to believe we know more than we do. Overconfidence reflects a tendency to act on incomplete information, intuitions, or hunches. Too much faith is placed in opinion instead of carefully gathered evidence.
Premature closure: the tendency to apply premature closure to the decision making process, accepting a diagnosis before it has been fully verified.
Representativeness: the representativeness heuristic drives the diagnostician toward looking for prototypical manifestations of disease: ‘‘If it looks like a duck, walks like a duck, quacks like a duck, then it is a duck.’’ Yet restraining decision-making along these pattern-recognition lines leads to atypical variants being missed.
Risk tolerance/aversion: Risk is the probability of the occurrence of an event. Risk tolerance is a measure of the degree of uncertainty that someone is willing to accept in respect of negative outcome.
Sunk costs: the more clinicians invest in a particular diagnosis, the less likely they may be to release it and consider alternatives. This cognitive distortion is more associated with financial considerations. It applied the concept of the disutility of giving up an object or idea which is greater than the utility associated with acquiring a new one. However, for the diagnostician, the investment is time and mental energy and, for some, ego may be a precious investment.

Table S1. Results of literature search according to the combination of MeSH terms

MESH Terms combination

	Medical errors	Cognition	Decision making	Physicians	Bias
Medical errors					
Cognition	7				
Decision making	75	766			
Physicians	462	350	5963		
Bias	14	710	1525	1455	

All combined with physicians

	Medical errors	Cognition	Decision making	Bias
Medical errors				
Cognition	3			
Decision making	20	27		
Bias	5	6	114	

All combined with case vignettes

	Medical errors	Cognition	Decision making	Bias
Medical errors				
Cognition	0			
Decision making	1	6		
Bias	1	5	25	

All combined with case vignettes + Physicians

	Medical errors	Cognition	Decision making	Bias
Medical errors				
Cognition	0			
Decision making	1	1		
Bias	1	1	9	
